# Performance of different adiposity measures for predicting left ventricular remodeling in Chinese hypertensive youth

**DOI:** 10.1038/s41598-021-00978-0

**Published:** 2021-11-09

**Authors:** Hong Cheng, Bo Xi, Junting Liu, Yinkun Yan, Jie Mi

**Affiliations:** 1 Department of Epidemiology, Capital Institute of Pediatrics, Beijing, 10020 China; 2 Department of Epidemiology, School of Public Health, Shandong University, Jinan, 250012 China; 3 Department of Non-Communicable Disease Management, Beijing Children’s Hospital, Capital Medical University, National Center for Children’s Health, Beijing, China

**Keywords:** Cardiology, Risk factors

## Abstract

There is no consistent conclusion on which adiposity measure is best to predict cardiovascular risk factors in youth. The present study aims to assess the performance of body mass index (BMI), waist circumference (WC), and waist-to-height ratio (WHtR) in predicting abnormal left ventricular structure in Chinese hypertensive youth. A total of 1180 youth aged 6–17 years with hypertension from the China Child and Adolescent Cardiovascular Health Study were included in this study. Logistic regression model, receiver operator characteristic (ROC) curve analysis and net reclassification improvement (NRI) method were used to assess performance of BMI, WC, and WHtR in predicting left ventricular hypertrophy (LVH) and left ventricular geometry (LVG). A 1-standard deviation increment in any of three indexes in predicting LVH and LVG were similar, e.g., with the odds ratios and 95% confidence intervals of 1.34 (1.16–1.55), 1.25 (1.08–1.45) and 1.40 (1.20–1.62), respectively. In addition, ROC analysis and NRI method confirmed the similar performance of three adiposity indexes in predicting LVH and LVG. In conclusion, BMI, WC and WHtR had similar performance in predicting abnormal left ventricular structure in Chinese hypertensive youth, but all three indexes had limited value in prediction. WHtR is a simple and convenient adiposity index for screening youth at high risk of target organ damage.

## Introduction

The prevalence of pediatric obesity is increasing globally during recent decades^1^. It is well established that childhood obesity is associated with not only short-term risk of target organ damage^2^, but also long-term risk of cardiovascular disease and premature mortality in adulthood^3^. Thus, it is important to identify youth with obesity to prevent related harmful health outcomes.

Body mass index (BMI) is a commonly used index for assessing pediatric obesity globally. However, BMI represents obese status in the whole body and it cannot distinguish fat mass from lean mass^4^. Additionally, the use of BMI defining obesity should be based on sex- and age- specific percentile cutoffs. Waist circumference (WC) represents the accumulation of fat mass at the abdomen, which is suggested to predict cardiovascular disease and type 2 diabetes better than BMI in adult population^5^. However, the use of WC also should be based on sex- and age-specific cutoffs.

Waist-to-height ratio (WHtR), which corrects height of a child, is another useful index to assess central obesity. A main advantage of WHtR is that it requires only one cutoff (0.50) to define central obesity^6^, which is simpler and more convenient to use than BMI and WC in pediatric clinical practice. However, although one recent meta-analysis showed that WHtR performs equally well as compared with BMI and WC in identification of children with cardiovascular risk factors^7^, it is unclear whether WHtR has the similar performance with BMI and WC in predicting abnormal left ventricular structure in pediatric population.

In the present study, we aimed to assess the performance of BMI, WC and WHtR in predicting left ventricular hypertrophy (LVH) and left ventricular geometry (LVG) in Chinese hypertensive youth.

## Methods

Our data were part of the China Child and Adolescent Cardiovascular Health (CCACH) Study, which is a large, nationwide study designed to examine cardiovascular risk factors among Chinese children aged 6–18 years. Detailed information on CCACH study has been described elsewhere^8^. In brief, a stratified cluster sampling method was used to select at least 600 school-age children per age group in each cluster (300 boys and 300 girls). Children and adolescents were recruited from six provinces (Changchun, Yinchuan, Beijing, Jinan, Shanghai and Chongqing) covering geographical areas in North, East, Northeast, Northwest and Southwest regions of China. A total of 44,793 children and adolescents with complete data on sex, age, weight, height, WC and blood pressure (BP) were included. Then, 1979 children and adolescents with elevated BP (≥ 95th percentile values for sex and age) on three different occasions were diagnosed as having hypertension. However, data on left ventricular structure were available for 1180 children and adolescents. Exclusion criteria were: (1) the inability to give informed consent; (2) use of any drug known to affect BP; (3) having chronic diseases and (4) pregnancy.

### Physical examination

In each of six centers, physical examination followed a standardized protocol by trained staff. Weight and height were measured in light clothes without shoes. Weight was measured twice to the nearest 0.1 kg, and height was measured twice to the nearest 0.1 cm. The two values of height or weight were averaged for data analysis. BMI was calculated as weight divided by the square of height (kg/m^2^).

BP was measured with an appropriate cuff size on the right arm in a sitting position using an oscillometric device (OMRON HEM-7012), which has been clinically validated in children and adolescents^9^. BP was measured three times on each of three occasions, and the last two readings were averaged for data analysis. According to the Chinese children and adolescents BP references^10^, elevated BP in children and adolescents was defined as BP equal to or above 95th percentile values by sex and age. Children with elevated BP on three separate occasions were defined to have hypertension according to the pediatric high BP guideline^11,12^.

### Echocardiographic assessment

Left ventricular structure was assessed using Sonographers performed Doppler ultrasonography device (Sonos 5500, Andover, MA, USA) with a 2.5–4.0 MHz probe. Left ventricular end-diastolic internal dimension, interventricular septal thickness and posterior wall thicknesses were measured. Left ventricular mass (LVM) was calculated using the Devereux`s formula LVM (g) = 0.8 × [1.04 × (left ventricular end-diastolic internal dimension + interventricular septal thickness + posterior wall thicknesses)^3^ − (left ventricular end-diastolic internal dimension)^3^] + 0.6^13^. Left ventricular mass index (LVMI) was calculated as LVM (in grams) divided by height (in meters)^2.7^ to correct for body size^14^. Left ventricular hypertrophy (LVH) was defined as LVMI ≥ age- and sex-specific 95th percentile values^15^. The relative wall thickness (RWT) was calculated as (posterior wall thicknesses + interventricular septal thickness) / left ventricular end-diastolic internal dimension. Increased RWT was defined using the cutoff of ≥ 0.36^16^. Based on LVMI and RWT, children and adolescents were then divided into four groups: normal geometry (normal LVMI and normal RWT), concentric remodeling (normal LVMI and increased RWT), eccentric hypertrophy (increased LVMI and normal RWT) and concentric hypertrophy (increased LVMI and increased RWT)^17^.

### Statistical analysis

All data analyses were performed using SAS 9.3 (SAS Institute, Cary, NC). BMI, WC and WHtR have different units, and it is incomparable between three adiposity indexes using the original units. Thus, we calculated sex- and age- specific z-scores based on the study sample. We calculated odds ratios (ORs) and 95% confidence interval (CIs) of LVH and LVG for BMI-z score, WC-z score and WHtR-z score, respectively, using binary and multinomial logistic regression models with adjustment for systolic and diastolic BP. We also assessed the performance of three adiposity indices in predicting LVH and LVG, respectively, using receiver operator characteristic (ROC) curve analysis and the area under the curve was calculated. Differences in area under the curves (AUCs) between any two indices were compared using Z test. Net reclassification improvement (NRI) was calculated to determine the extent to which adiposity indexes (BMI, WC and WHtR) improves the predictive ability for LVH or LVG^18,19^. We performed subgroup analyses by sex and age group. *P* < 0.05 indicates statistical significance.

### Patient and public involvement

No patients or the public were involved in the design, or conduct, or reporting, or dissemination plans of this research. Refer to the Methods section for further details.

### Patient consent for publication

All the subjects’ parents provided signed informed consent.

### Ethics approval

The study was approved by the Research Ethics Committee of the Capital Institute of Paediatrics of China (2,012,062). We confirm that all methods were carried out in accordance with relevant guidelines and regulations.

## Results

**Figure 1:**
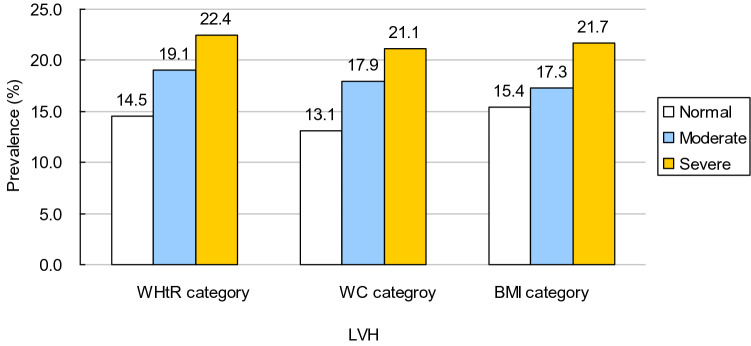
Prevalence of LVH across three categories (normal, moderate, and severe) of each of three adiposity indices. Notes Normal, moderate, and severe status for WHtR was defined using 0- < 0.45, 0.45- < 0.50, and ≥ 050, respectively; for WC defined using < 75th percentile, 75th—< 90th percentile, and ≥ 90th percentile, respectively; for BMI defined using < 85th percentile, 85th—< 95th percentile, and ≥ 95th percentile, respectively.

A total of 1180 children and adolescents with complete data on adiposity indexes and left ventricular structure were included in the final analysis. Table 1 presents the characteristics of study sample by sex. BMI, WC and WHtR were higher in boys than in girls (all *p* < 0.0001). However, there was no difference in LVMI, RWT, and prevalence of LVH and LVG between boys and girls (all *p* > 0.05). The prevalence of LVH increased significantly across three categories (normal, moderate, and severe) of each of three adiposity indices (Fig. 1).

**Table 1:** Characteristics of study population.

	Total (n = 1180)	Boys (n = 796)	Girls (n = 384)	*P* value
Age, years	11.8 (3.3)	12.0 (3.2)	11.2 (3.4)	0.0002
Height, cm	156.8 (18.1)	160.2 (18.4)	149.8 (15.2)	< 0.0001
Weight, cm	61.0 (23.6)	66.1 (23.9)	50.3 (18.8)	< 0.0001
BMI, kg/m^2^	23.8 (5.8)	24.9 (5.7)	21.7 (5.3)	< 0.0001
WC, cm	76.8 (15.3)	80.1 (15.2)	69.8 (12.8)	< 0.0001
WHtR	0.49 (0.08)	0.50 (0.08)	0.47 (0.07)	< 0.0001
SBP, mmHg	129.3 (10.6)	131.5 (10.6)	124.9 (9.3)	< 0.0001
DBP, mmHg	72.7 (7.3)	71.9 (7.5)	74.3 (6.8)	< 0.0001
LVMI, g/m^2.7^	29.3 (24.3–36.9)	29.4 (24.4–37.3)	29.2 (24.0–36.0)	0.4369
LVH, %	18.9	17.7	21.4	0.1345
RWT, cm	0.32 (0.29–0.35)	0.32 (0.29–0.35)	0.32 (0.29–0.35)	0.5453
LVG, %				0.3920
Normal geometry	72.3	72.9	71.0	
Concentric remodeling	8.8	9.4	7.6	
Eccentric hypertrophy	6.9	6.4	7.8	
Concentric hypertrophy	12.0	11.3	13.6	

Continuous variables are presented as mean (SD) if they are in normal distribution or median (P25-P75) if not.

BMI, body mass index; WC, waist circumference; WHtR, waist-to-height ratio; SBP, systolic blood pressure; DBP, diastolic blood pressure; LVMI, left ventricular mass index; LVH, left ventricular hypertrophy; RWT, relative wall thickness; LVG, left ventricular geometry.

The increased risk of LVH or LVG for 1-standard deviation (SD) increment of three adiposity indices were similar (Table 2). For example, the ORs and 95% CIs of LVH for BMI-z score, WC-z score and WHtR-z score were 1.34 (1.16–1.55), 1.25 (1.08–1.45) and 1.40 (1.20–1.62), respectively. There were significant interactions between each of three adiposity indexes and sex or age group (*P* < 0.05), and then we performed subgroup analyses. The associations were more pronounced in boys than in girls for three adiposity indexes (Table S1). For LVH and eccentric hypertrophy, the associations were more pronounced in adolescents than in children; while for concentric remodeling, it was reversed (Table S1).

**Table 2:** ORs and 95% CIs of LVH and LVG according to different adiposity indexes in hypertensive youth.

	OR (95% CI)	*P* value
**LVH**		
BMI-Z score	1.34 (1.16–1.55)	< 0.0001
WC-Z score	1.25 (1.08–1.45)	0.0027
WHtR-Z score	1.40 (1.20–1.62)	< 0.0001
**Concentric remodeling**		
BMI-Z score	1.17 (0.96–1.44)	0.1220
WC-Z score	1.27 (1.03–1.56)	0.0224
WHtR-Z score	1.22 (0.99–1.50)	0.0609
**Eccentric hypertrophy**		
BMI-Z score	1.41 (1.13–1.75)	0.0020
WC-Z score	1.22 (0.97–1.54)	0.0825
WHtR-Z score	1.41 (1.13–1.77)	0.0027
**Concentric hypertrophy**		
BMI-Z score	1.34 (1.13–1.60)	0.0011
WC-Z score	1.33 (1.11–1.60)	0.0017
WHtR-Z score	1.44 (1.20–1.72)	< 0.0001

LVH, left ventricular hypertrophy; LVG, left ventricular geometry; BMI, body mass index; WC, waist circumference; WHtR, waist-to-height ratio.

Logistic regression models were adjusted for systolic and diastolic blood pressure.

Performance of BMI-z score, WC-z score and WHtR-z score to predict LVH or LVG was also similar using ROC analysis or NRI method (Table 3). For example, AUCs were 0.580, 0.561 and 0.592 for BMI-z score, WC-z score and WHtR-z score, respectively. The difference in AUCs between three adiposity indexes was not significant (*p* > 0.05). In addition, the NRI was also not significant for WC-z score or WHtR-z score, as compared with BMI-z score (*p* > 0.05). The subgroup analyses by sex and age are shown in Table S2.

**Table 3:** Performance of BMI, WC and WHtR to predict LVH and LVG in hypertensive youth.

	AUC (95% CI)	*P* value	NRI, %	*P* value
**LVH**				
BMI-Z score	0.58 (0.54–0.62)	…	…	…
WC-Z score	0.56 (0.52–0.61)	0.061	-2.4	0.307
WHtR-Z score	0.59 (0.55–0.63)	0.215	1.6	0.489
**Concentric remodeling**				
BMI-Z score	0.55 (0.48–0.61)	…	…	…
WC-Z score	0.57 (0.51–0.63)	0.038	4.6	0.117
WHtR-Z score	0.56 (0.49–0.62)	0.392	2.1	0.239
**Eccentric hypertrophy**				
BMI-Z score	0.58 (0.50–0.65)	…	…	…
WC-Z score	0.54 (0.47–0.61)	0.050	-6.8	0.215
WHtR-Z score	0.58 (0.52–0.65)	0.747	0.4	0.930
**Concentric hypertrophy**				
BMI-Z score	0.59 (0.54–0.64)	…	…	…
WC-Z score	0.59 (0.53–0.64)	0.734	-0.2	0.930
WHtR-Z score	0.61 (0.56–0.66)	0.090	5.7	0.018

AUC, area under the curve; LVH, left ventricular hypertrophy; LVG, left ventricular geometry; BMI, body mass index; WC, waist circumference; WHtR, waist-to-height ratio; NRI, net reclassification improvement.

## Discussion

### Main findings

To our knowledge, this is the first study investigating the associations of three different adiposity indexes (i.e., BMI, WC and WHtR) with left ventricular remodeling in Chinese hypertensive youth. Our findings suggest that the performance of three adiposity indexes is similar in predicting presence of LVH or LVG. It seems that WHtR, as a simple and convenient adiposity indexes, can replace with BMI and WC that require sex- and age- specific percentile cutoffs.

### Compared with previous studies

The findings on which adiposity index performs best in predicting cardiovascular risk factors in children and adolescents have been inconsistent. A recent meta-analysis including 34 studies based on general pediatric population indicated that WHtR performed equally well compared with BMI and WC in predicting cardiovascular risk factors including hyperglycaemia, elevated blood pressure, dyslipidemia and metabolic syndrome^7^. To our knowledge, no studies have assessed the performance of WHtR as compared to BMI for predicting abnormal left ventricular structure in hypertensive youth. A study of 156 patients aged 2–20 years (without structural heart disease) found that WHtR was positively associated with both LVM and LVMI^20^. Analysis of 1723 visits of 593 children (mean age: 12.2 years) with chronic kidney disease showed that WHtR performed better than BMI for predicting not only cardiovascular risk profile (including abnormal levels of HDL-C, TG and non-HDL-C) but also higher LVMI^21^. In all, all three studies above showed that WHtR has similar or better performance than BMI in predicting higher LVMI. Since WHtR only requires static cutoffs (without consideration of a child’s age and sex) to define central obesity, WHtR is convenient to use in clinical practice, as well as in other settings such as in schools for school nurses and at home for parents, respectively, to monitor central obesity status of a child.

We found that the overall ROC values of three adiposity indices were not high enough (0.55–0.60), suggesting pediatric obesity alone has limited predictive ability for abnormal left ventricular structure. In addition, other predictors such as hypertension, dyslipidemia, and hyperglycemia may play additional important roles in predicting left ventricular remodeling. We also found that the ROC values for predicting LVH and eccentric hypertrophy were much lower in children aged 6–12 years (0.51–0.54 for LVH, 0.53–0.60 for eccentric hypertrophy) than in adolescents aged 13–17 years (0.64–0.68 for LVH, 0.72–0.76 for eccentric hypertrophy), suggesting that adiposity indices are not useful to predict LVH and eccentric hypertrophy in children but may be useful in adolescents. In contrast, for predicting concentric remodeling, the ROC values were much higher in children aged 6–12 years (0.68–0.72) than in adolescents aged 13–17 years (0.50–0.52). These results above suggest that adiposity indices have different predictive ability to determine different LV left ventricular remodeling patterns in different age groups of youths.

As for the optimal cutoff of WHtR for screening cardiovascular risk factors in Asian children and adolescents, the findings have also been inconsistent. But most studies support that the cutoff of WHtR should be lower than 0.50^22–24^. A cross-sectional study including 16,914 Chinese children and adolescents aged 7–17 years demonstrated that the optimal cutoffs of WHtR should be 0.47 for boys and 0.45 for girls^23^. Another cross-sectional study including 3057 Korean adolescents aged 10–19 years showed that the optimal cutoffs of WHtR should be 0.44 for boys and 0.43 for girls^24^.

### Strengths and limitations

This is the first study to examine the associations of BMI, WC and WHtR with left ventricular remodeling in Chinese youth with hypertension. Our study has four limitations. First, the cross-sectional design of this study impeded us to make casual inference. Second, we assessed the performance of three adiposity indexes in hypertensive youth, and the generalizability of our findings to general healthy youth should be made with caution. Third, although body fat composition might be superior to three adiposity indices studied in current study, information on body fat composition is unavailable. Fourth, more novel and sensitivity cardiac markers such as LV diastolic parameters e', contractility s' or LV filling pressure E/e' are widely used in clinical studies. However, these data are unavailable currently. In addition, left atrium (LA)/ left ventricle (LV) index is useful for assessing sub-clinical LA/LV dysfunction. However, LA was not measured in the dataset.

## Conclusion

Our study shows that three adiposity indexes (BMI, WC and WHtR) have similar performance in predicting abnormal left ventricular structure in Chinese hypertensive youth. However, it should be noted that each of three adiposity indexes has limited prediction value. The more simplicity and convenience of WHtR as compared with BMI may be useful in screening youth at high risk of target organ damage.

## Supplementary Information

Supplementary Information.
