# Supplementary material for: Performance of different adiposity measures for predicting left ventricular remodeling in Chinese hypertensive youth
Source: Sci Rep. 2021 Nov 9;11:21943. doi: 10.1038/s41598-021-00978-0 (PMC8578612; doi:10.1038/s41598-021-00978-0)
Supplement: Supplementary file 1 — Supplementary Information. [file 41598_2021_978_MOESM1_ESM.doc]

**Table S1: ORs and 95% CIs of LVH and LVG for different adiposity indexes in hypertensive youth by sex and age**

|  | **Prevalence,%** | **OR (95% CI)** | | |
| --- | --- | --- | --- | --- |
|  |  | BMI-z score | WC-z score | WHtR-z score |
| **LVH** |  |  |  |  |
| Boys | 17.7 | 1.42 (1.18-1.70) | 1.35 (1.12-1.62) | 1.45 (1.21-1.75) |
| Girls | 21.4 | 1.20 (0.94-1.53) | 1.09 (0.85-1.40) | 1.30 (1.01-1.66) |
| 6-12 years | 23.0 | 1.06 (0.87-1.28) | 1.05 (0.86-1.27) | 1.17 (0.96-1.41) |
| 13-17 years | 14.4 | 1.92 (1.50-2.46) | 1.61 (1.26-2.05) | 1.85 (1.44-2.38) |
|  |  |  |  |  |
| **Concentric remodeling** |  |  |  |  |
| Boys | 9.4 | 1.22 (0.96-1.54) | 1.39 (1.08-1.79) | 1.30 (1.02-1.67) |
| Girls | 7.6 | 1.08 (0.73-1.60) | 1.05 (0.72-1.54) | 1.04 (0.71-1.53) |
| 6-12 years | 5.1 | 1.76 (1.27-2.44) | 2.00 (1.40-2.86) | 1.84 (1.29-2.64) |
| 13-17 years | 12.9 | 0.96 (0.74-1.26) | 1.05 (0.81-1.36) | 1.02 (0.79-1.33) |
|  |  |  |  |  |
| **Eccentric hypertrophy** |  |  |  |  |
| Boys | 6.4 | 1.40 (1.06-1.84) | 1.21 (0.90-1.63) | 1.34 (1.00-1.79) |
| Girls | 7.8 | 1.43 (0.99-2.05) | 1.22 (0.85-1.75) | 1.52 (1.06-2.20) |
| 6-12 years | 7.7 | 0.93 (0.67-1.28) | 0.83 (0.59-1.15) | 1.04 (0.76-1.41) |
| 13-17 years | 6.0 | 2.71 (1.84-3.99) | 2.17 (1.49-3.14) | 2.40 (1.63-3.55) |
|  |  |  |  |  |
| **Concentric hypertrophy** |  |  |  |  |
| Boys | 11.3 | 1.48 (1.19-1.85) | 1.54 (1.22-1.94) | 1.61 (1.28-2.02) |
| Girls | 13.6 | 1.11 (0.81-1.51) | 1.03 (0.76-1.40) | 1.18 (0.87-1.60) |
| 6-12 years | 15.4 | 1.21 (0.97-1.52) | 1.28 (1.01-1.61) | 1.33 (1.06-1.68) |
| 13-17 years | 8.5 | 1.54 (1.13-2.10) | 1.36 (1.00-1.83) | 1.60 (1.17-2.18) |

OR, odds ratio; CI, confidence interval; LVH, left ventricular hypertrophy; LVG, left ventricular geometry; BMI, body mass index; WC, waist circumference; WHtR, waist-to-height ratio.

Logistic regression models were adjusted for systolic blood pressure and diastolic blood pressure.

**Table S2:** **Performance of BMI, WC and WHtR to predict LVH and LVG in hypertensive youth by sex and age**

|  | AUC (95% CI) | *P* value | NRI, % | *P* value |
| --- | --- | --- | --- | --- |
| **LVH** |  |  |  |  |
| **Boys** |  |  |  |  |
| BMI-z score | 0.595 (0.541-0.649) | … | … | … |
| WC-z score | 0.572 (0.516-0.629) | 0.0578 | -0.5 | 0.8551 |
| WHtR-z score | 0.597 (0.544-0.651) | 0.8046 | 0.1 | 0.9598 |
|  |  |  |  |  |
| **Girls** |  |  |  |  |
| BMI-z score | 0.557 (0.488-0.627) | … | … | … |
| WC-z score | 0.543 (0.475-0.612) | 0.4453 | 4.4 | 0.4292 |
| WHtR-z score | 0.587 (0.520-0.653) | 0.1268 | 3.5 | 0.4167 |
|  |  |  |  |  |
| **6-12 years** |  |  |  |  |
| BMI-z score | 0.519 (0.464-0.575) | … | … | … |
| WC-z score | 0.511 (0.454-0.567) | 0.5473 | -6.0 | 0.5320 |
| WHtR-z score | 0.543 (0.488-0.597) | 0.1130 | 5.6 | 0.0540 |
|  |  |  |  |  |
| **13-17 years** |  |  |  |  |
| BMI-Z score | 0.677 (0.613-0.740) | … | … | … |
| WC-Z score | 0.643 (0.575-0.711) | 0.0071 | -7.3 | 0.0695 |
| WHtR-Z score | 0.672 (0.609-0.735) | 0.7010 | -1.5 | 0.7322 |
|  |  |  |  |  |
| **Concentric remodeling** |  |  |  |  |
| **Boys** |  |  |  |  |
| BMI-Z score | 0.554 (0.480- 0.627) | … | … | … |
| WC-Z score | 0.585 (0.511- 0.659) | 0.0248 | 9.5 | 0.0841 |
| WHtR-Z score | 0.572 (0.501- 0.644) | 0.1377 | 1.4 | 0.7263 |
|  |  |  |  |  |
| **Girls** |  |  |  |  |
| BMI-Z score | 0.525 (0.405-0.645) | … | … | … |
| WC-Z score | 0.531 (0.420-0.643) | 0.7351 | 1.8 | 0.9266 |
| WHtR-Z score | 0.512 (0.399-0.626) | 0.6358 | -7.5 | 0.2944 |
|  |  |  |  |  |
| **6-12 years** |  |  |  |  |
| BMI-Z score | 0.697 (0.595-0.799) | … | … | … |
| WC-Z score | 0.716 (0.620-0.811) | 0.3049 | 2.1 | 0.6755 |
| WHtR-Z score | 0.682 (0.584-0.780) | 0.5402 | -6.9 | 0.3023 |
|  |  |  |  |  |
| **13-17 years** |  |  |  |  |
| BMI-Z score | 0.518 (0.445-0.592) | … | … | … |
| WC-Z score | 0.507 (0.434-0.581) | 0.8819 | -9.2 | 0.4436 |
| WHtR-Z score | 0.501 (0.429-0.574) | 0.8170 | -7.5 | 0.5227 |
|  |  |  |  |  |
| **Eccentric hypertrophy** |  |  |  |  |
| **Boys** |  |  |  |  |
| BMI-Z score | 0.575 (0.484- 0.666) | … | … | … |
| WC-Z score | 0.525 (0.433- 0.616) | 0.0397 | -13.3 | 0.1519 |
| WHtR-Z score | 0.560 (0.475- 0.645) | 0.5214 | 5.8 | 0.2740 |
|  |  |  |  |  |
| **Girls** |  |  |  |  |
| BMI-Z score | 0.578 (0.457- 0.698) | … | … | … |
| WC-Z score | 0.554 (0.440- 0.668) | 0.486 | -6.3 | 0.3083 |
| WHtR-Z score | 0.623 (0.517- 0.730) | 0.2263 | 0.3 | 0.9506 |
|  |  |  |  |  |
| **6-12 years** |  |  |  |  |
| BMI-Z score | 0.556 (0.461-0.651) | … | … | … |
| WC-Z score | 0.597 (0.504-0.689) | 0.2074 | -0.5 | 0.9501 |
| WHtR-Z score | 0.526 (0.438-0.614) | 0.3851 | -12.5 | 0.1665 |
|  |  |  |  |  |
| **13-17 years** |  |  |  |  |
| BMI-Z score | 0.756 (0.674-0.839) | … | … | … |
| WC-Z score | 0.719 (0.636-0.802) | 0.0390 | -4.8 | 0.6315 |
| WHtR-Z score | 0.733 (0.651-0.814) | 0.1259 | -5.8 | 0.5568 |
|  |  |  |  |  |
| **Concentric hypertrophy** |  |  |  |  |
| **Boys** |  |  |  |  |
| BMI-Z score | 0.616 (0.551- 0.681) | … | … | … |
| WC-Z score | 0.615 (0.545- 0.685) | 0.9098 | -1.7 | 0.6982 |
| WHtR-Z score | 0.632 (0.566- 0.697) | 0.1594 | 2.5 | 0.5775 |
|  |  |  |  |  |
| **Girls** |  |  |  |  |
| BMI-Z score | 0.551 (0.471- 0.631) | … | … | … |
| WC-Z score | 0.541 (0.461- 0.621) | 0.6140 | -4.3 | 0.4978 |
| WHtR-Z score | 0.567 (0.489- 0.646) | 0.4208 | -2.9 | 0.4397 |
|  |  |  |  |  |
| **6-12 years** |  |  |  |  |
| BMI-Z score | 0.576 (0.513-0.639) | … | … | … |
| WC-Z score | 0.585 (0.520-0.649) | 0.5025 | 7.4 | 0.0614 |
| WHtR-Z score | 0.595 (0.532-0.658) | 0.1446 | 5.3 | 0.2020 |
|  |  |  |  |  |
| **13-17 years** |  |  |  |  |
| BMI-Z score | 0.617 (0.532-0.703) | … | … | … |
| WC-Z score | 0.592 (0.497-0.687) | 0.1317 | -2.9 | 0.3938 |
| WHtR-Z score | 0.630 (0.543-0.716) | 0.4491 | -1.0 | 0.8283 |

LVH, left ventricular hypertrophy; LVG, left ventricular geometry; BMI, body mass index; WC, waist circumference; WHtR, waist-to-height ratio; AUC, area under the curve; NRI, net reclassification improvement; CI, confidence interval.
